# Urinary Gluten Peptide Determination: Results Are Results, Even When They Contradict Aprioristic Expectations

**DOI:** 10.14309/ctg.0000000000000472

**Published:** 2022-03-16

**Authors:** Chiara Monachesi, Anil K. Verma, Giulia N. Catassi, Elisa Franceschini, Simona Gatti, Rosaria Gesuita, Elena Lionetti, Carlo Catassi

**Affiliations:** 1 Celiac Disease Research Laboratory, Polytechnic University of Marche, Ancona, Italy;; 2 Division of Pediatrics, DISCO Department, Polytechnic University of Marche, Ancona, Italy;; 3 Center of Epidemiology and Biostatistics, Polytechnic University of Marche, Ancona, Italy;; 4 Mucosal Immunology and Biology Research Center, Division of Pediatric Gastroenterology and Nutrition, Massachusetts General Hospital, Boston, USA.

We thank Sousa et al. for carefully reading our article entitled “Determination of Urinary Gluten Immunogenic Peptides to Assess Adherence to the Gluten-Free Diet: A Randomized, Double-Blind, Controlled Study” and for giving us the opportunity to clarify some aspects of this study (1,2).

As for the analytical procedures, we confirm that urinary gluten immunogenic peptide (GIP) was quantified in urine samples with positive qualitative results of the iVYCHECK GIP Urine using the iVYCHECK Reader (Biomedal S.L., Seville, Spain), strictly following the manufacturer's protocol during all steps of sample preparation and test procedure. All laboratory procedures were performed in our specialized Celiac Disease Research Laboratory by 2 of us (C.M. and A.K.V.).

Since we took several precautions to avoid confounding factors in a real-life scenario, as described in the Methods section, we do not see any reason to consider our results “inconsistent” simply because urinary GIP was often unexpectedly positive (in subjects on the gluten-free diet [GFD] or after the placebo challenge) or negative (after the gluten challenge). Inconsistency may indeed be an intrinsic feature of this test. Nor it is a cause for concern the observation that our results are at variance with “over a dozen studies from multiple groups reporting the validity, the utility, and reliability of the GIP test in the monitoring of the GFD,” for the humble reason that no previous studies investigated in depth the relationship between the dose of ingested gluten and the result of the urinary GIP test.

As suggested by our substudy B, the high rate of urinary GIP positivity in subjects on the GFD (34%) may be interpreted as a background noise caused by traces of gluten (<10 mg), which are still tolerable and allowed in the standard GFD. Interestingly, the possibility that a negligible amount of gluten may cause urinary GIP positivity has recently been reported by Silvester et al. (3). This finding is a significant limitation of the test that may occasionally become an advantage in the follow-up of subjects on the gluten contamination elimination diet, i.e., a zero-gluten diet. The high rate of false negative urinary GIP in subjects challenged with gluten (32%), likely related to interindividual variations in the metabolism of gluten peptides, is even more worrisome because it may generate an unjustified feeling of adherence to the GFD in celiac patients actually ingesting significant amounts of gluten. Likewise, poor sensitivity of the iVYCHECK GIP assay in stool has been recently reported in healthy subjects on the GFD challenged with either 50 mg or 2 g of gluten (4). In general, lack of a positive correlation between the dose of ingested gluten and the result of urinary GIP in the range of 10 mg to 1 g of gluten, i.e., the amount of gluten that is more commonly involved in involuntary transgressions to the GFD (5–6), suggests that the determination of urinary GIP is not a reliable method to assess the compliance to the GFD, at least by the currently available commercial laboratory kit.

According to Karl Popper's principles of scientific research, our study awaits confirmation or confutation by other experimental investigations, possibly conducted by independent researchers with no direct interest in the commercialization of items/devices that are the object of the study.

## CONFLICTS OF INTEREST

**Guarantor of the article:** Carlo Catassi, MD, MPH.

**Specific author contributions:** C.M., A.K.V., C.C.: writing-original draft, writing-review and editing; G.N.C., E.F., S.G., R.G., E.L.: writing-review and editing. All authors read and approved the final version of the manuscript.

**Financial support:** None to report.

**Potential competing interests:** C.C. is a scientific consultant of Dr. Schaer Food. All other authors declare no conflicts of interest.

## References

[1] Monachesi C Verma AK Catassi GN . Determination of urinary gluten immunogenic peptides to assess adherence to the gluten-free diet: A randomized, double-blind, controlled study. Clin Transl Gastroenterol 2021;12(10):e00411. 34613954 10.14309/ctg.0000000000000411 PMC8500619

[2] Sousa C Comino I Cebolla A . Controls and result interpretations in studies of urine gluten peptide determinations. Clin Transl Gastroenterol 2022;13(1):e00456. 35060931 10.14309/ctg.0000000000000456 PMC8806359

[3] Silvester JA Comino I Rigaux LN . Exposure sources, amounts and time course of gluten ingestion and excretion in patients with coeliac disease on a gluten-free diet. Aliment Pharmacol Ther 2020;52:1469–79. 32981131 10.1111/apt.16075 PMC7780203

[4] Coto L Sousa C Cebolla A. Individual variability in patterns and dynamics of fecal gluten immunogenic peptides excretion after low gluten intake. Eur J Nutr 2022:1–17. 34993643 10.1007/s00394-021-02765-z PMC8739026

[5] Silvester JA Comino I Kelly CP . Most patients with celiac disease on gluten-free diets consume measurable amounts of gluten. Gastroenterology 2020;158:1497–9. 31866245 10.1053/j.gastro.2019.12.016 PMC7103503

[6] Monachesi C Verma AK Catassi GN . Quantification of accidental gluten contamination in the diet of children with treated celiac disease. Nutrients 2021;13:190. 10.3390/nu13010190 PMC7827942 33435453

